# Supplementary material for: Combining Asian and European genome-wide association studies of colorectal cancer improves risk prediction across racial and ethnic populations
Source: Nat Commun. 2023 Oct 2;14:6147. doi: 10.1038/s41467-023-41819-0 (PMC10545678; doi:10.1038/s41467-023-41819-0)
Supplement: Supplementary file 1 — Supplementary Information [file 41467_2023_41819_MOESM1_ESM.pdf]

Supplementary Methods and Results

Test and Validation Data Sets

The Minority GWAS Japanese study (MG-JPN)

The Japanese samples (n =4,655) were identified from the Multiethnic Cohort study (MEC), the Colorectal Cancer Family Registry (CCFR), the Japan Public Health Center-based Prospective study (JPHC) and three case–control studies conducted in Hawaii (CR2&3) and in Fukuoka and Nagano, Japan. Comprehensive details on the participants subjects, genotyping and standard quality control (QC) procedures have been reported in[1] and are summarized in **Table 1.**

The Minority GWAS African American study (MG-AA)

The African American samples (n = 6,597) were identified from the MEC, CCFR, the Southern Community Cohort Study (SCCS), the MD Anderson Cancer Center, the University of North Carolina CanCORS study (UNC-CanCORS) and Rectal Cancer Study (UNC-Rectal), and from the Prostate, Lung, Colorectal and Ovarian Cancer Screening (PLCO) Trial. The details on the participants, genotyping, QC procedures were published in [1] and are summarized in Table 1.

The Hispanic Colorectal Cancer Study (HCCS)

The Hispanic Colorectal Cancer Study (HCCS) is a population-based study of individuals self-identified as Hispanic with a diagnosis of CRC. Cases were identified from the California Cancer Registry or directly from local hospitals in the Los Angeles region [LAC + USC County Hospital and University of Southern California (USC) Norris Comprehensive Cancer Center]. All men and women over 21 years of age with a first-time diagnosis of CRC (ICD-O-3 codes: C18–C21) after January 1, 2008, were eligible for participation. Risk factor/dietary questionnaires, pathology reports and saliva samples (for genotyping) were collected using methodologies developed in the Colon Cancer Family Registry and the Multiethnic Cohort (MEC). The present study includes 950 cases recruited into the HCCS who were born in Mexico (42.3%), the USA (31.4%), Central/South America (16.6%), Cuba or the Caribbean Islands (1.8%) or Europe (0.4%). This study was approved by the USC Institutional Review Board and the California Committee for the Protection of Human Subjects, and all participants provided written

informed consent. The detailed descriptions of the participants, genotyping, QC procedure were given in [2] and are summarized in Table 1.

Multiethnic Cohort Sstudy (MEC)

The Multiethnic Cohort Study (MEC) [2] is a large prospective cohort study that includes people from various ethnic groups, including Hispanic/Latino (HL) primarily from California and mainly, Los Angeles. Between 1993 and 1996, participants returned a self-administered baseline questionnaire that obtained general demographic, medical and risk factor information. The MEC used state driver's license files as the primary source to identify study participants in California. Surnames were used to identify HL individuals because race/ethnicity was not available in driver's license files.

In the cohort, incident cancer cases were identified annually through cohort linkage to population-based Surveillance, Epidemiology and End Results cancer registries in Los Angeles County as well as to the California State cancer registry in the same manner as in the HCCS. All men and women over age 21 with a first-time diagnosis of CRC (ICD-O-3 codes: C18–C21) were included as eligible cases. The current study used questionnaire data and DNA samples derived from whole blood or buccal cells for 661 HL prevalent or incident CRC cases born in the USA (57.8%), Mexico (27.7%), Central/South America (9.7%), Cuba or the Caribbean Islands (4.4%), or Europe (0.2%). Individuals without a diagnosis of CRC were used as controls (n=2,106). All MEC controls self-reported being born in the USA (52.2%), Mexico (34.3%), or Central/South America (13.2%). This study includes 661 cases and 2,106 controls.

This study was approved by the University of Southern California and the University of Hawaii Institutional Review Boards, and all participants provided informed consent.

Cancer Prevention Study II (CPS II)

The CPS II Nutrition cohort is a prospective study of cancer incidence and mortality in the United States, established in 1992 and described in detail elsewhere [3]. At enrolment, participants completed a mailed self-administered questionnaire including information on demographic, medical, diet, and lifestyle factors. Follow-up questionnaires to update exposure information and to ascertain newly diagnosed cancers were sent biennially starting in 1997. Reported cancers were verified through medical records,

state cancer registry linkage, or death certificates. The Emory University Institutional Review Board approved all aspects of the CPS II Nutrition Cohort.

Two independent sets of CPS II cases and matched controls were included in the training data set described above. A third independent set of 804 cases and 908 and sex- and age-matched controls were included in this validation effort.

Basque-colon cohort

Basque-colon cohort included 873 participants with colon cancer, biobanked between 2009-2019. Participants were diagnosed and/or treated according to usual clinical practice in the Donostia University Hospital, San Sebastian, Spain, including follow-up to this date. Controls (n = 945) came from the blood donor biobank of the same geographical area.

Genetic Epidemiology Research on Adult Health and Aging (GERA) Cohort

The Genetic Epidemiology Research on Adult Health and Aging (GERA) resource is a cohort of more than 100,000 participants from Kaiser Permanente Medical Care Plan, Northern California Region (KPNC), Research Program on Genes, Environment and Health (RPGEH). Genome-wide genotyping was targeted for this cohort to enable large-scale genome-wide association studies by linkage to comprehensive longitudinal clinical data derived from extensive KPNC electronic health record databases. The cohort is multi-ethnic, with ~20% minority representation (African American, East and Southeast Asian, and Hispanic/Latinx or mixed), and the remaining 80% non-Hispanic white. For this project, four ancestry-specific arrays were designed based on the Affymetrix Axiom Genotyping System. Imputation was performed using 1000 Genomes data on an array-wise basis.

Construction of this cohort began in 2007, when a six-page survey was mailed to 1.9 million individuals, age 18 and older, who had been previously enrolled health plan members for at least two years. This survey was designed to ascertain data on demographic and lifestyle characteristics, including race/ethnicity, education, income, marital status, and family history for 35 selected conditions, diet and physical activity, smoking and alcohol consumption, and reproductive history and health. In July 2008, approximately 400,000 survey respondents were asked to sign a consent form authorizing broad use of their survey data, longitudinal electronic health record data, and biospecimens in conducting research on genetic and environmental factors associated with health and disease. Those who provided consent were

mailed saliva DNA collection (Oragene) kits. In 2009, over 40,000 men ages 45 to 69 years, who were KPNC health plan members and had enrolled in the California Men's Health Study (CMHS) in 2002–2003, were similarly asked to provide saliva samples and added to expand the GERA cohort. At CMHS enrollment, men completed mailed surveys to ascertain data on demographic and lifestyle factors, akin to that of GERA.

In total, 110,266 consenting participants who provided saliva samples were selected for genotyping. All racial and ethnic minority participants with saliva samples (n = 20,935, 19%) were included to maximize diversity. Four custom Affymetrix Axiom arrays were designed for genotyping, one for each major ancestral group represented in the GERA cohort: European, African, East Asian, and Latinx. As detailed elsewhere [4], the selected number of SNPs and SNP content varied by array in order to maximize coverage of the whole genome, along with common and low frequency SNPs specific to race/ethnicity and known SNPs associated with disease phenotypes. Details on the calling and quality control have been described in [5].

A personal history of cancer was determined from cancer registry and electronic health record data, medical record coding and electronic pathology report coding. A family history of CRC was ascertained through integrating data from baseline surveys and electronic health records (i.e., diagnosis codes, family history documentation). All study participants provided written informed consent, and the study was approved by the KPNC Institutional Review Board. As a cohort unselected on any disease phenotype, GERA participants were not asked to engage in specific medical or screening tests for research purposes. Therefore, although the majority (69%) of GERA participants were age 55 and older at baseline, most, but not all, have undergone screening for colorectal cancer, either by fecal immunochemical testing (FIT) or endoscopy (sigmoidoscopy or colonoscopy). At their baseline questionnaire, 70% were up to date for colorectal cancer screening.

Colorectal cancer status was determined for study participants from their initiation of KPNC health plan membership by linkage to the KPNC Cancer Registry, which captures data on all participants with newly diagnosed cancers at KPNC facilities that adheres to the National Cancer Institute's Surveillance, Epidemiology, and End Results (SEER) Program standards. The observed time was defined from the age of initial KPNC enrollment to the earliest of age at CRC diagnosis, death or end of follow-up (until

December 31, 2016), truncated age to 95, and the CRC incidence is measured from 2007 to 2016. While stratifying participants based on family history, we classified them as yes for those who have positive family history of CRC and no with those who have no information on family history. After quality control, we included 77,012 non-Hispanic White (1,401 cases; 75,611 controls), 7,370 Asian (96 cases; 7,274 controls), 3,159 African American (56 cases; 3,103 controls) and 6,660 Latinx/Hispanic (70 cases; 6,590 controls) participants. CRC status was determined from cancer-registry data.

Electronic Medical Records and Genomics (eMERGE)

The eMERGE network has been developing a unified genome-wide single-nucleotide variant (SNV) genotype array-based association platform for analysis of electronic medical record (EMR)-derived phenotypes for approximately 10 years[6–8]. In the first phase, eMERGE 1, discovery efforts were based on the Illumina 660k genotype array with ~20,000 participants being enrolled through five medical centers. In eMERGE 2 ~30,000 more individuals with high-density genotype data were ascertained resulting in analyses with ~50,000 individuals. In eMERGE 3, genotype and clinical EMR data of ~33,000 additional participants have been added to the resources available for analysis. The case control status for CRC was defined by an algorithm based on ICD9 codes 153 -153.9, 154 - 154.2,154.8 and ICD10 codes C18-C18.9, C19, C20, C21-C21.2, C21.8 (https://phekb.org/phenotype/colorectal-cancer-crc). After removal of low-call-rate samples (>2% missingness) and duplicated samples, the data set resulted in 83,717 unique imputed adult participants based on the eMERGE subject IDs from 77 imputation batches. The details of the imputation and quality control are given in [9] .

Methods

PRS-CSx

We used PRS-CSx[10], a recently developed Bayesian polygenic modeling method, to construct the ancestry-specific PRS while leveraging other ancestral GWAS summary statistics. It [10] is a Python based command line tool that integrates GWAS summary statistics and external LD reference panels from multiple populations to improve cross-population polygenic prediction. PRS-CSx is an extension of the Bayesian polygenic prediction method PRS-CS with Bayesian regression and continuous shrinkage prior.

PRS-CSx couples genetic effects across populations via a shared continuous shrinkage prior, enabling more accurate effect size estimation by sharing information of summary statistics between populations, while incorporating linkage disequilibrium (LD) diversity across populations. For SNP j in population k, PRS-CSx uses a continuous shrinkage prior on its effect size β_{jk}, which can be represented as global-local scale mixtures of normals:

$$\beta_{jk} \sim N\left(0, \frac{\sigma_k^2}{N_k} \psi_j\right), \quad \psi_j \sim Gamma\,(a, \delta_j), \delta_j \sim Gamma\,(b, \Phi) \qquad (1)$$

where σ_k^2 and N_k are variance and the number of individuals in population k respectively, Φ is a global shrinkage parameter shared across all SNPs that models the overall sparseness of the genetic architecture, and ψ_j is a local, SNP-specific shrinkage parameter that is adaptive to marginal GWAS associations. By assigning a gamma–gamma hierarchical prior on ψ_j (specifically, the Strawderman–Berger prior with a = 1 and b = 1/2), the marginal prior density of β_{jk} has a sizable amount of mass near zero to impose strong shrinkage on small noisy signals, and, in the meantime, heavy Cauchy-like tails to avoid over-shrinkage of truly nonzero effects.

The shared prior allows for correlated but varying effect size estimates across populations, retaining the flexibility of the modeling framework. We used pre-computed LD reference panels based on the UK Biobank data as provided with the PRS-CSx tool. The PRS-CSx included ~1.2 million HapMap3 variants in the PRS calculation.

LDpred2

LDpred [11]uses a Bayesian approach for SNP selection and shrinkage for PRS with a spike-and-slab prior, based on an LD matrix and GWAS summary statistics. We used the updated version LDPred2 implemented in the R package bigsnpr. LDpred has been demonstrated to provide higher predictive performance, particularly with large GWAS sample size as in our study and addresses previous instability issues. Further, the use of a larger window of 3cM (using genetic distance rather than number of bases) improves performance when causal variants are in regions with long-range LD, such as HLA regions. In particular, LDpred assumes the following model for SNP effect sizes β_j,

$$\beta_j \sim N(0, \frac{h^2}{Mp}) \quad \text{with probaility } p \qquad (2)$$

0, otherwise.

where p is the proportion of causal variants, M the number of variants and h^2 the (SNP) heritability. The parameters were estimated using Gibbs sampler.

LDpred2[12] is an extension of the LDpred model which derives the PRS based on GWAS summary statistics and a matrix of correlation between genetic variants[12]. Here the summary statistics was obtained from the meta-analysis of the Asian and European GWAS. The correlation matrix was calculated as a weighted average of ancestry-specific LD matrices estimated based on a subset of Asian (nAsian = 7,370) and European (nEuropean = 61,493) samples, where the weight is proportional to the sample sizes of Asian and European GWAS in the summary statistics. We used the LDpred-grid model, which uses a grid of hyper parameters SNP heritability, h^2, and proportion of causal variants, p. LDpred2 estimates the heritability from constrained LD score regression. The heritability estimates for 1,016,596 SNPs retained in the PRS was 0.10. We used the validation data set, GERA cohort, to obtain the optimal value for the proportion of causal variants, p and heritability, h^2.

PRS Calculation

As described in [13], the time that takes to calculate PRS may vary depending on the computation power, data set format and the tools selected for the analysis. We calculated the PRS using allelic dosages in PLINK 2[14]. Using nodes with ~300GB RAM for 20 cores, 200 SNPs took less than 24 milliseconds whereas 1 million SNPs took less than 72 milliseconds to generate PRS for each individual.

Supplemental Table 1: AUC estimates (95% confidence interval) for European-centric PRS, known loci PRS, PRS-CSx and LDpred2

Case and Ethnicity	Studies	% of SNPs	$n_{case}/n_{control}$	EUR-centric PRS	Known Loci PRS	PRS-CSx	LDPred2
**Asian**	GERA	100%	96/7,274	0.619 (0.547-0.689)	0.587 (0.536-0.638)	0.64 (0.58-0.69)	0.627 (0.579-0.675)
	MG-JPN	100%	2, 324/ 2,331	0.587 (0.571-0.603)	0.601 (0.586-0.618)		0.632 (0.616-0.644)
**Black or African American**	GERA	100%	56/3,103	0.526 (0.419-0.620)	0.503 (0.444-0.613)		0.546 (0.456-0.636)
	MG-AA	99%	1856/ 4741	0.577 (0.560-0.596)	0.580 (0.560-0.596)		0.593 (0.576-0.612)
	eMERGE#	100%	42/4,025	0.574 (0.480-0.660)	0.590 (0.500-0.690)		0.620 (0.520-0.720)
**Latinx or Hispanic**	GERA	100%	70/6590	0.584 (0.511-0.656)	0.587 (0.513-0.661)		0.614 (0.547-0.681)
	Hispanic GWAS	100%	1611/2106	0.612 (0.594-0.634)	0.614 (0.592-0.632)		0.635 (0.615-0.655)
**Non-Hispanic White**	GERA	100%	1,401/75,611	0.638 (0.624-0.652)	0.615 (0.600-0.629)	0.64 (0.62-0.65)	0.653 (0.638-0.668)
	BCC	100%	873/945	0.617 (0.547-0.687)	0.610 (0.550-0.670)		0.645 (0.580-0.710)
	CPS II	100%	804/908	0.619 (0.591-0.647)	0.603 (0.576-0.630)		0.634 (0.606-0.6624)
	eMERGE#	99.7%	573/37,641	0.620 (0.600-0.640)	0.610 (0.54-0.68)		0.640 (0.610-0.670)
	^ Number of Variants Available / Number of Variants in Score (%) of LDpred2<br>*# AUC is adjusted for age, sex and 10 PCs*						

Supplemental Table 2: Odds Ratios (OR), 95% confidence interval (95% CI) and two-sided p-values for PRS per SD for all and stratified by family history and age.

	Asian		Black or African American		Latinx or /Hispanic		Non-Hispanic White		
	GERA	**MG-JPN**	***GERA***	**MG-AA**	***GERA***	***Hispanic GWAS***	***GERA***	***BCC***	***CPS II***
	**OR (95% CI)<br>p-value**	**OR (95% CI)<br>p-value**	**OR (95% CI)<br>p-value**	**OR (95% CI)<br>p-value**	**OR (95% CI)<br>p-value**	**OR (95% CI)<br>p-value**	**OR (95% CI)<br>p-value**	**OR (95% CI)<br>p-value**	**OR (95% CI)<br>p-value**
**PRS per SD**	1.58<br>(1.29-1.94)<br>1.3e-05	1.64<br>(1.55-1.73)<br><2.0e-18	1.25<br>(0.96-1.64)<br>0.090	1.40<br>(1.33-1.48)<br><2.0e-18	1.45<br>(1.16-1.80)<br>1.3e-03	1.63<br>(1.52-1.75)<br><2.0e-18	1.70<br>(1.62-1.79)<br><2.0e-18	1.72<br>(1.43-2.07)<br>7.93e-09	1.58<br>(1.44-1.74)<br><2.0e-18
**Family History**									
**No**	1.58<br>(1.24-2.00)<br>1.8e-04	1.64<br>(1.46-1.83)<br>7.00e-17	1.30<br>(0.97-1.1.73)<br>0.08	1.73<br>(1.34-2.22)<br>2.03e-05	1.53<br>(1.20-1.96)<br>6.7e-04	1.65<br>(1.52-1.79)<br><2.0e-18	1.69<br>(1.60-1.79)<br><2.0e-18		1.60<br>(1.44-1.78)<br>2.00e-18
**Yes**	1.30<br>(0.84-2.01)<br>0.229	1.49<br>(1.20-1.84)<br>3.4e-04	1.05<br>(0.535-2.05)<br>0.89	1.23<br>(1.04-1.46)<br>1.23e-02	0.998<br>(0.54-1.82)<br>0.995	1.18<br>(0.91-1.53)<br>0.206	1.54<br>(1.38-1.72)<br>4.58e-14		1.34<br>(1.00-1.77)<br>4.55e-02
**Age**									
<50	13 cases and 1,295 controls	174 cases and 166 controls	4 cases and 382 controls	293 cases and 98 controls	13 cases and 1288 controls	258 cases and 2 controls	59 cases and 5,127 controls	20 cases and 676 controls	--
	1.69<br>(0.954- 2.98)<br>0.07	1.92<br>(1.50- 2.45)<br>2.13e-07	1.36<br>(0.484- 3.84)<br>0.56	1.52<br>(1.17-1.97)<br>1.58e-03	1.17<br>(0.683- 2.00)<br>0.569	--	1.93<br>(1.50- 2.48)<br>3.22e-07	1.60<br>(1.00- 2.54)<br>0.04	
50 to 60	29 cases and 1,390 controls	516 cases and 494 controls	12 cases and 539 controls	478 cases 822 controls	14 cases and 1318 controls	434 cases and198 controls	196 cases and 9,743 controls	91 cases and 212 controls	41 cases and 50 controls
	1.68<br>(1.14-2.48)<br>0.009	1.87<br>(1.62-2.16)<br>1.17e-17	1.51<br>(0.824-2.76)<br>0.182	1.53<br>(1.36-1.72)<br>2.11e-12	1.80<br>(1.08-2.99)<br>0.023	2.22<br>(1.80-2.74)<br>3.52e-16	1.80<br>(1.57- 2.06)<br>1.41e-17	1.58<br>(1.22-2.06)<br>6.01e-04	1.72<br>(1.02-2.88)<br>0.042
60 to 70	21 cases and 2,064 controls	832 cases and 877 controls	19 cases and 872 controls	630 cases and 1,661 controls	17 cases and 1,710 controls	495 cases and 990 controls	377 cases and 19,831 controls	213 cases and 56 controls	301 cases and 337 controls
	1.63<br>(1.06- 2.50)<br>0.024	1.57<br>(1.42- 1.74)<br><2.0e-18	1.87<br>(1.145-3.06)<br>0.012	1.40<br>(1.27-1.54)<br>1.88e-11	1.67<br>(1.03- 2.69)<br>0.036	1.58<br>(1.42-1.77)<br>3.81e-16	1.92<br>(1.74- 2.12)<br><2.0e-18	2.18<br>(1.49- 3.20)<br>6.69e-05	1.72<br>(1.46-2.01)<br>3.05e-11
70 to 80	21 cases and 1,437 controls	652 cases and 693 controls	15 cases and 806 controls	380 cases and 1,824 controls	17 cases and 1,379 controls	329 cases and 760 controls	426 cases and 21,932 controls	--	346 cases and 398 controls
	1.52<br>(0.955- 2.40)<br>0.077	1.57<br>(1.40- 1.76)<br>6.91e-15	0.84<br>(0.51-1.38)<br>0.494	1.33<br>(1.19- 1.549)<br>3.46e-07	1.252<br>(0.768- 2.04)<br>0.367	1.49<br>(1.30-1.69)<br>1.78e-09	1.66<br>(1.52-1.82)<br><2.0e-18	--	1.49<br>(1.29-1.72)<br>8.24e-08
>80	12 cases and 1,088 controls	101 cases and 150 controls	6 cases and 504 controls	75 cases and 336 controls	9 cases and 895 controls	95 cases and 156 controls	343 cases and 18,978 controls	--	116 cases and 123 controls
	1.27<br>(0.682- 2.38)<br>0.477	1.74<br>(1.31- 2.30)<br>1.08e-04	0.87<br>(0.36-2.06)<br>0.745	1.37<br>(1.05- 177)<br>1.86e-02	1.5864<br>(0.83- 2.99)<br>0.16	1.57<br>(1.18-2.10<br>2.0e-03	1.42<br>(1.28- 1.58)<br>1.90e-11		1.50<br>(1.15-1.95)<br>2.79e-03

Supplemental Figure 1: ROC curves: ROC curves of PRS for colorectal cancer (CRC), stratified by race and ethnicity.

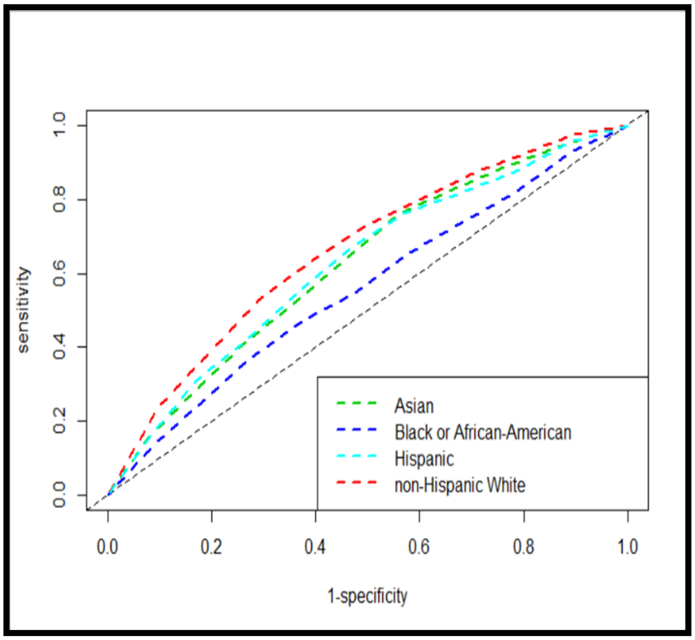

Supplemental Figure 2: Distribution of PRS for GERA cohort. A) PRS distributions varied across the racial and ethnic groups, B) PRS distribution after ancestry adjustment.

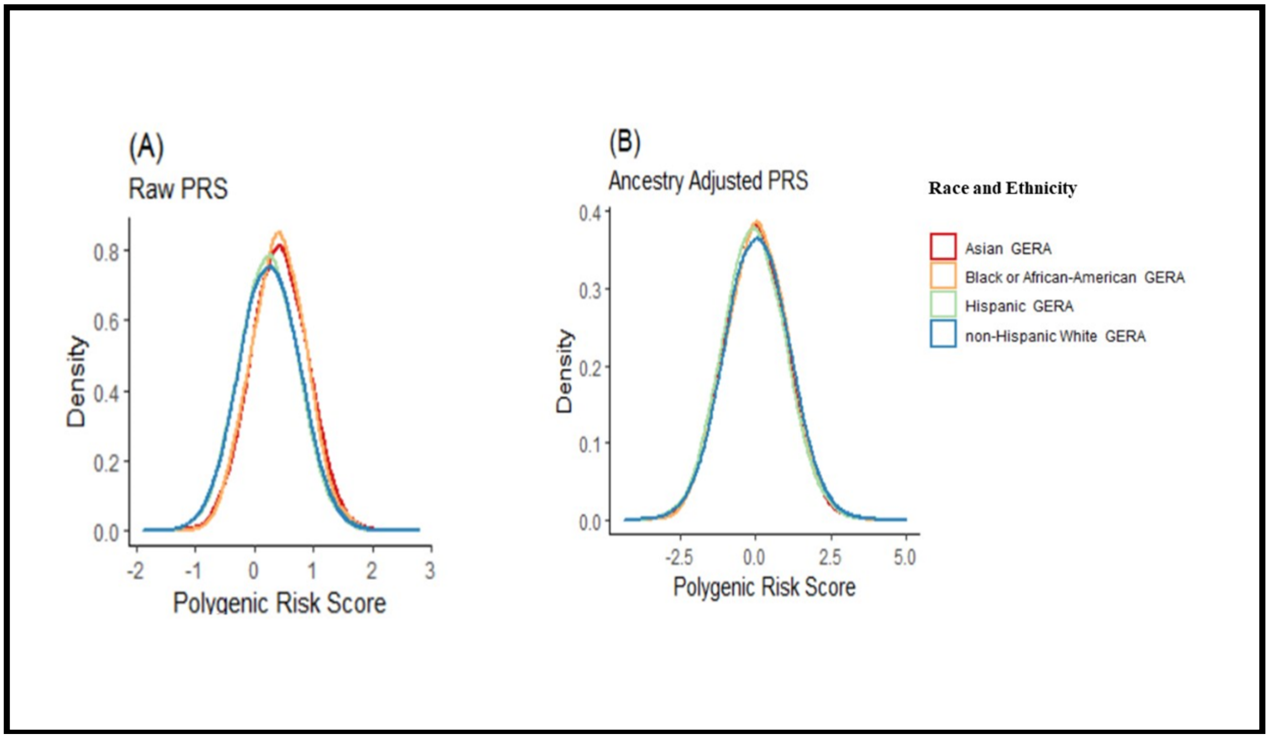

Supplemental Figure 3: Distribution of PRS for all other validation studies except for the GERA cohort. A) PRS distributions varied across the racial and ethnic groups, B) PRS distribution after ancestry adjustment, C) Additional mean adjustment for the MG-JP that has a different imputation panel.

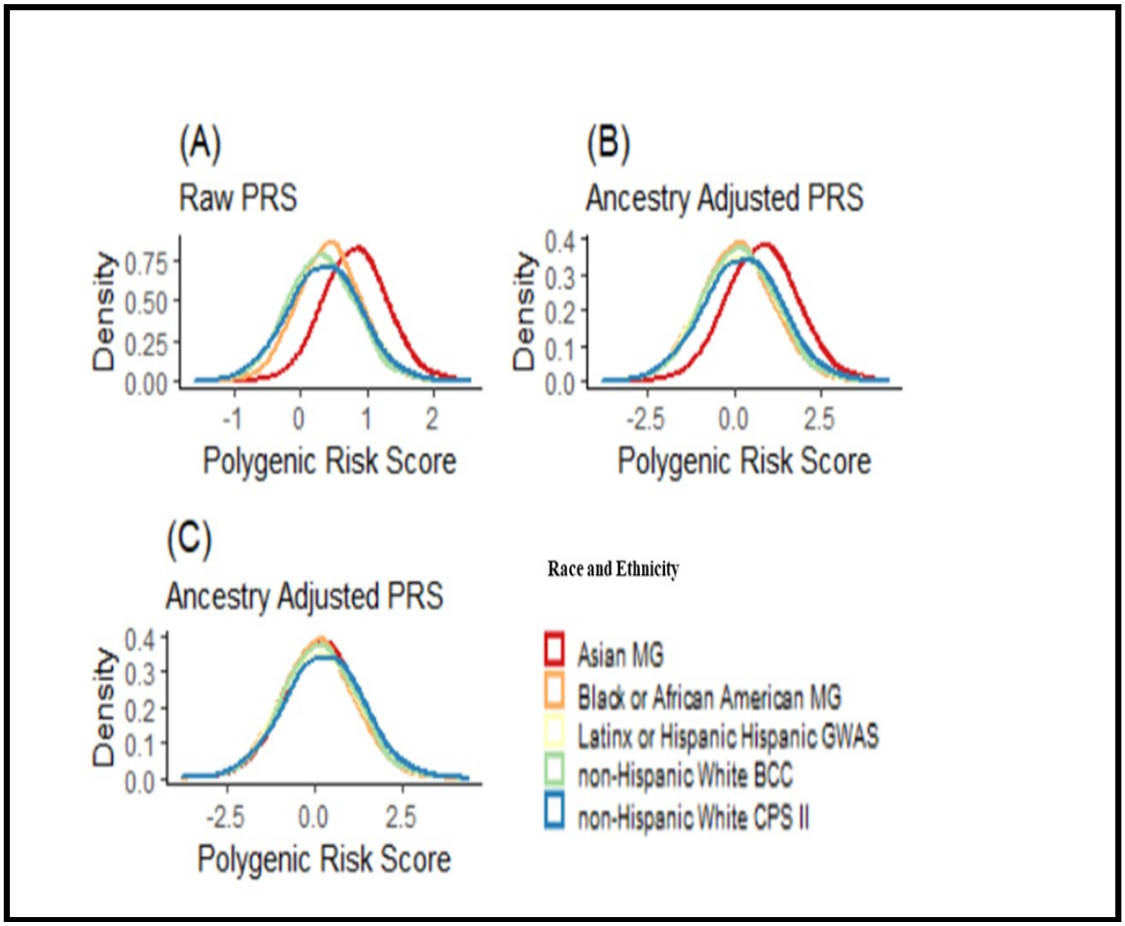

Supplemental Figure 4: Distribution of the ancestry adjusted PRS: Distribution of the ancestry adjusted PRS for GERA cohort, N=7,370; 3,159; 6,660; 77,012 and for other validation data sets N=4,655; 6,597; 3,717; 3,530, of unrelated samples by Asian, Black/African-American, Hispanic and non-Hispanic White, respectively. PRS is the single cross-ancestry Asian-European PRS based on LDpred2.

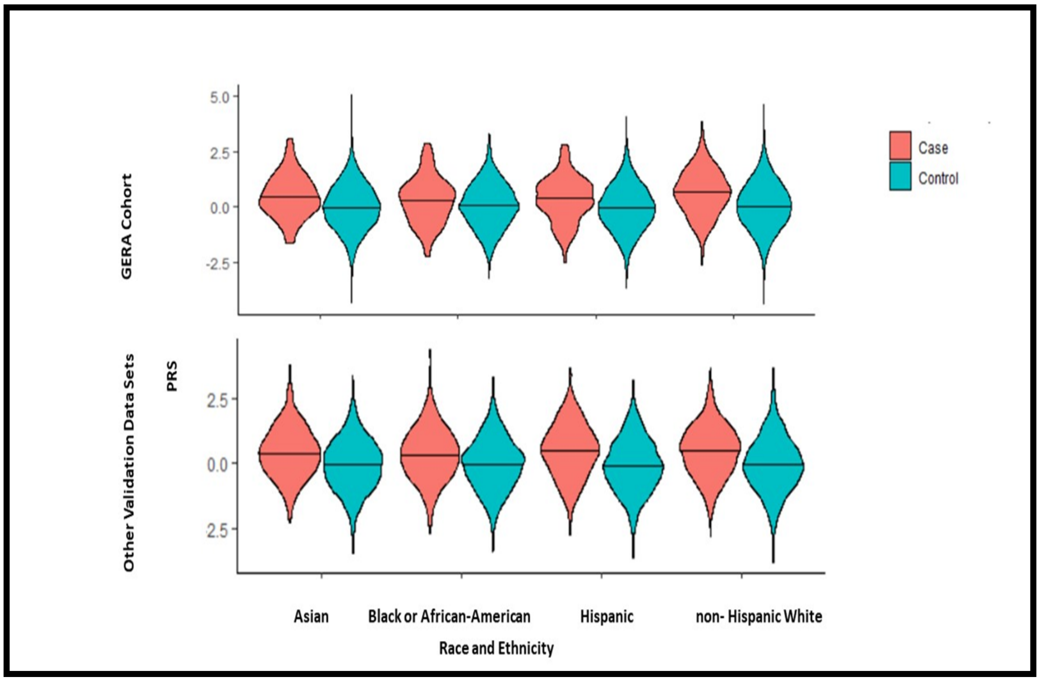

Supplemental Figure 5: Decision-Curves stratified by ancestral groups. Decision curves for the model based on family history and PRS, model based on family history alone, intervene for all, and intervene for none, in GERA study participants with age 40-49.

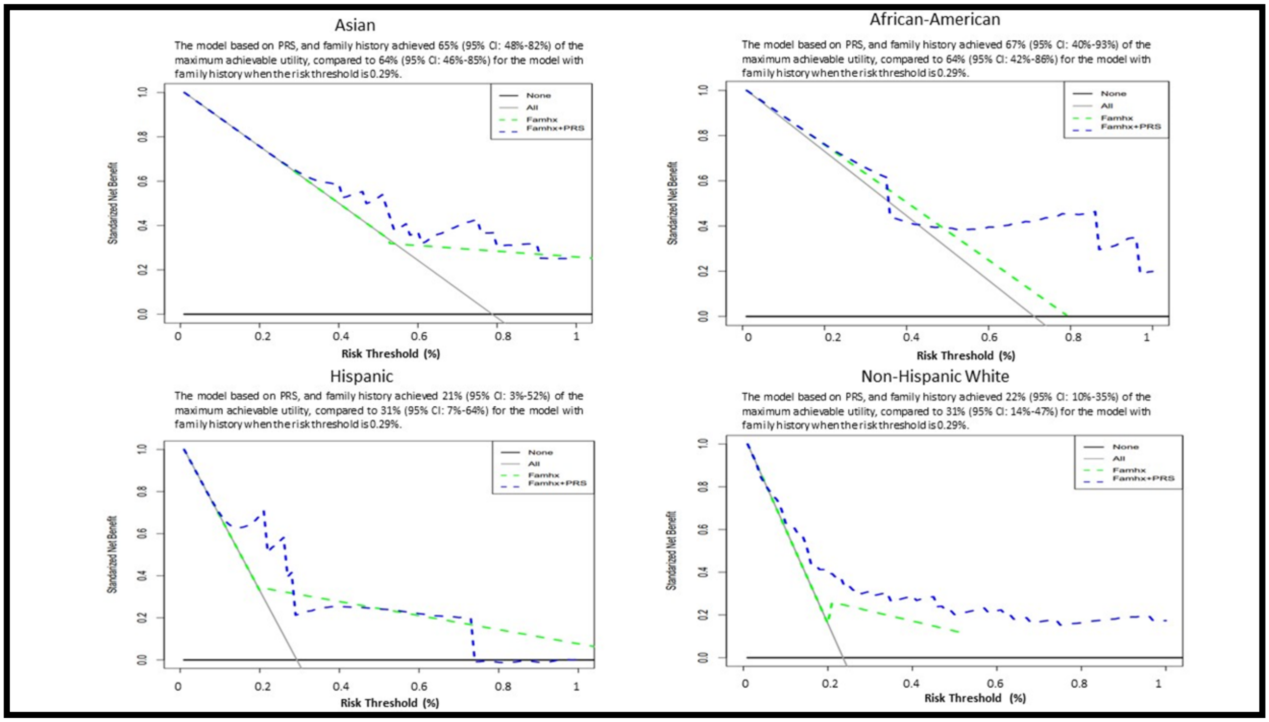

Supplemental Figure 6: Decision-Curves for the participants with age 50-59. Decision-Curves for the model based on family history and PRS, model based on family history alone, intervene for all, and intervene for none, in GERA study. The model based on PRS, and family history achieved 52.4% (95% CI: 45%-60%) of the maximum achievable utility, compared to 45.1% (95% CI: 35%-54%) for the model with family history only (p-value = 0.045) when the risk threshold is 0.29%.

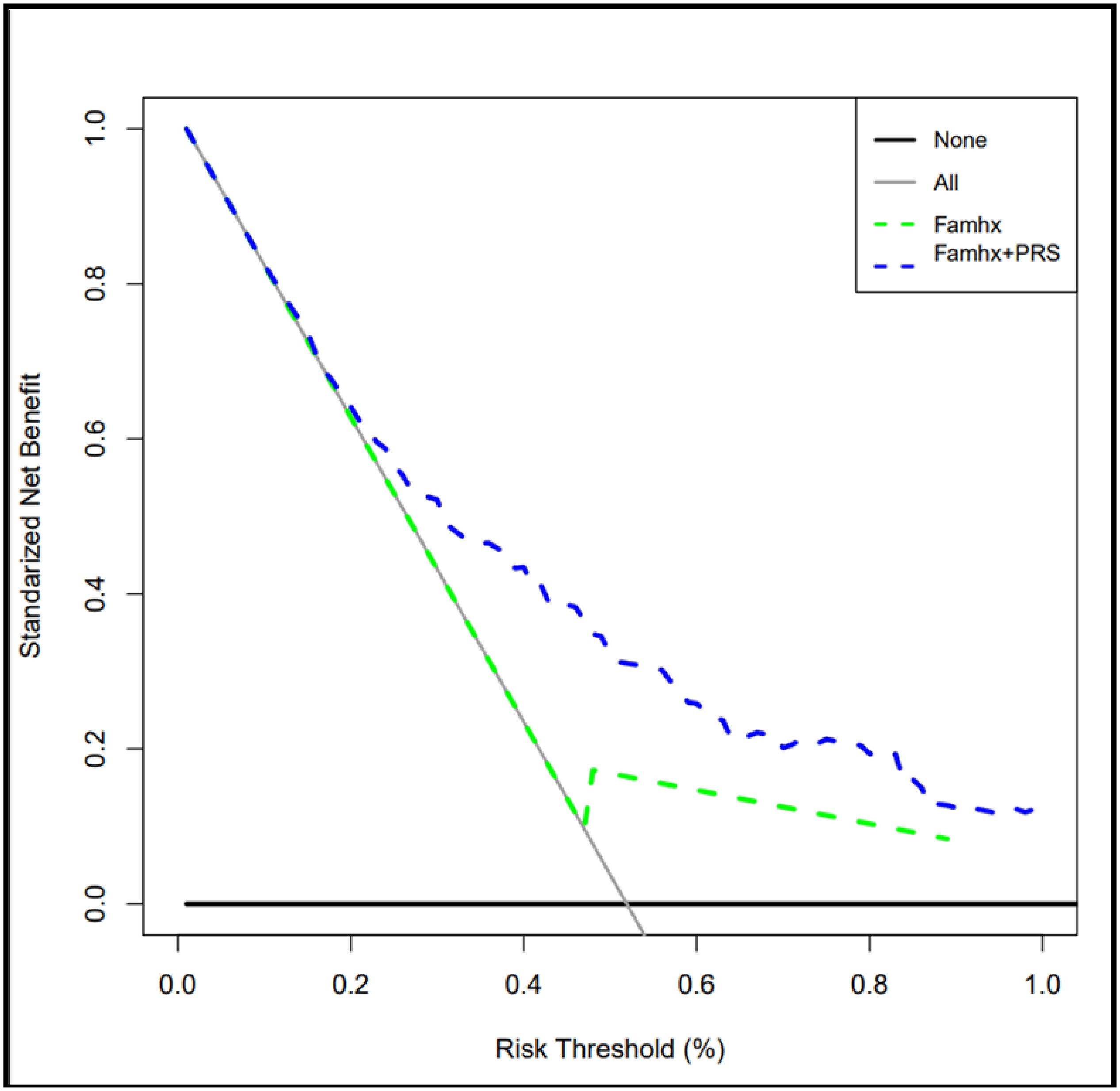

Supplemental Figure 7: Intervention Avoided 100 Individuals: Unnecessary interventions avoided per 100 individuals with age 40-49 at risk threshold 0.29% for models-based family (FamHx) only and FamHx + polygenic risk scores (PRS).

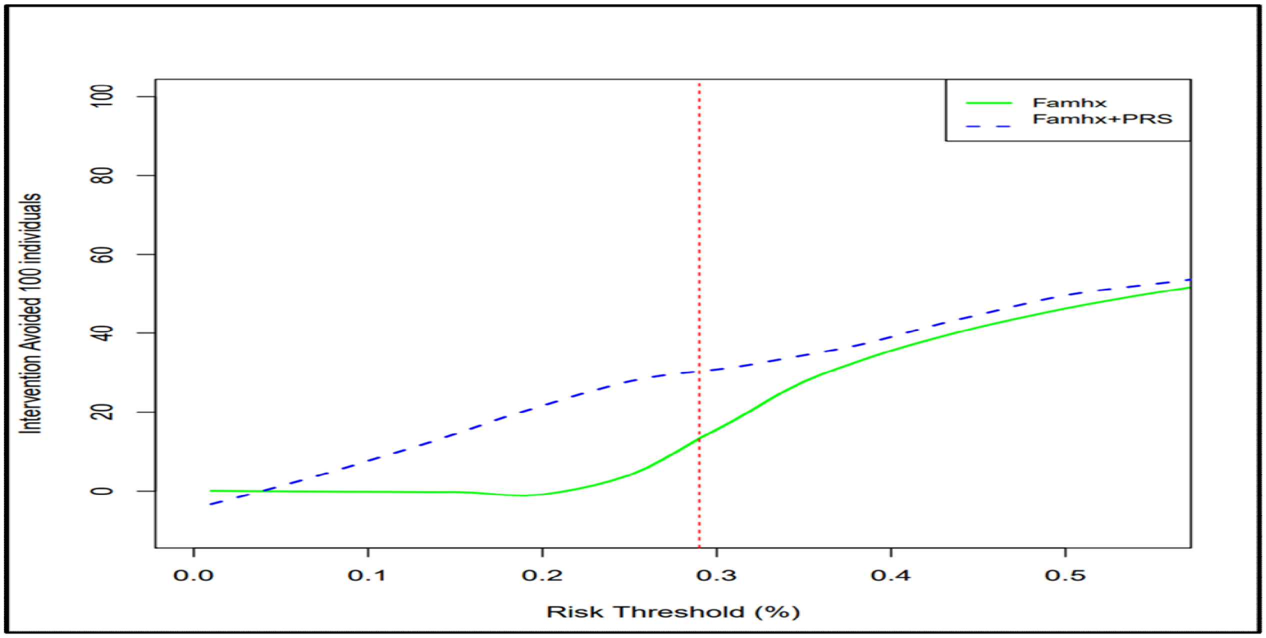

Supplemental Figure 8: Kaplan-Meier (K-M) Plots stratified by family history for different racial and ethnic groups: K-M cumulative incidence curves of a) Family history positive (FamHx+) and b) No Family History (FamHx-) for a) Black or African American (AA), b) Asian, c) Hispanic, and d) Non-Hispanic White (NHW) groups.

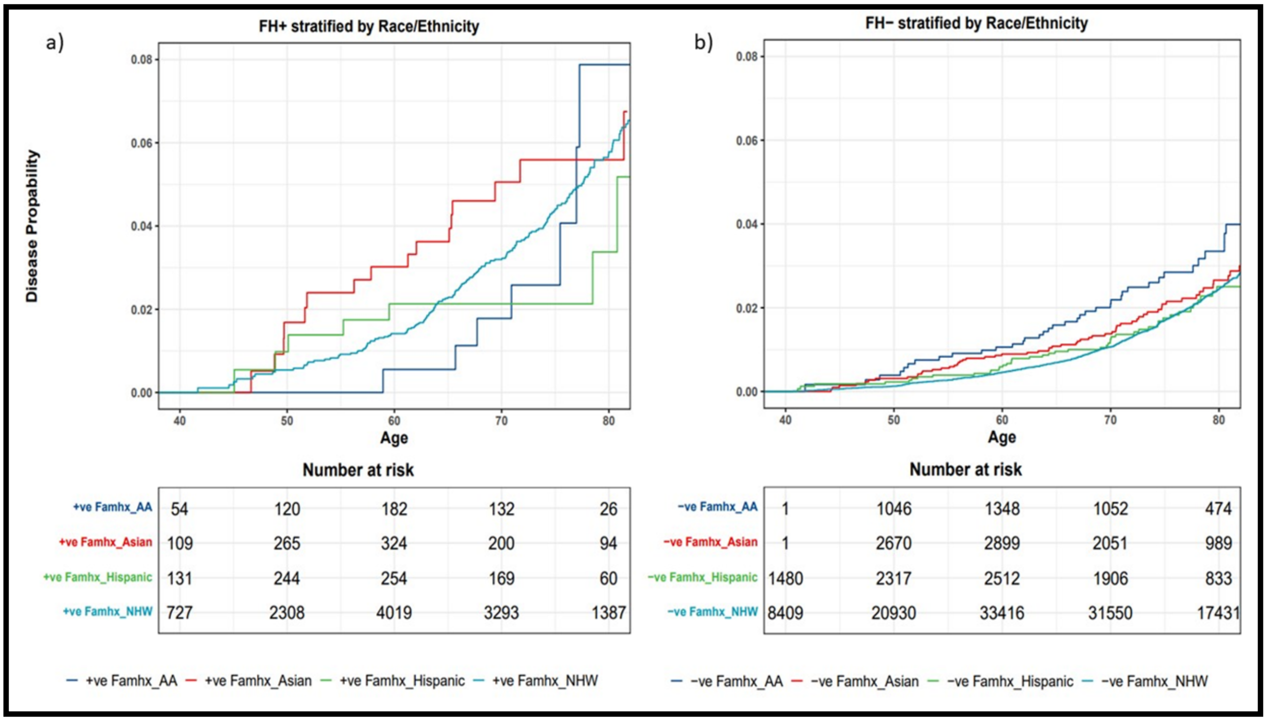

Supplemental Figure 9: Kaplan-Meier (K-M) Plots stratified by quantiles of PRS for different ancestral groups. K-M cumulative incidence curves by quantiles of PRS, top 5%, top 25%, 25-75%, bottom 25%, and bottom 5% for a) Black or African American, b) Asian, c) Hispanic, and d) Non-Hispanic White (NHW) groups.

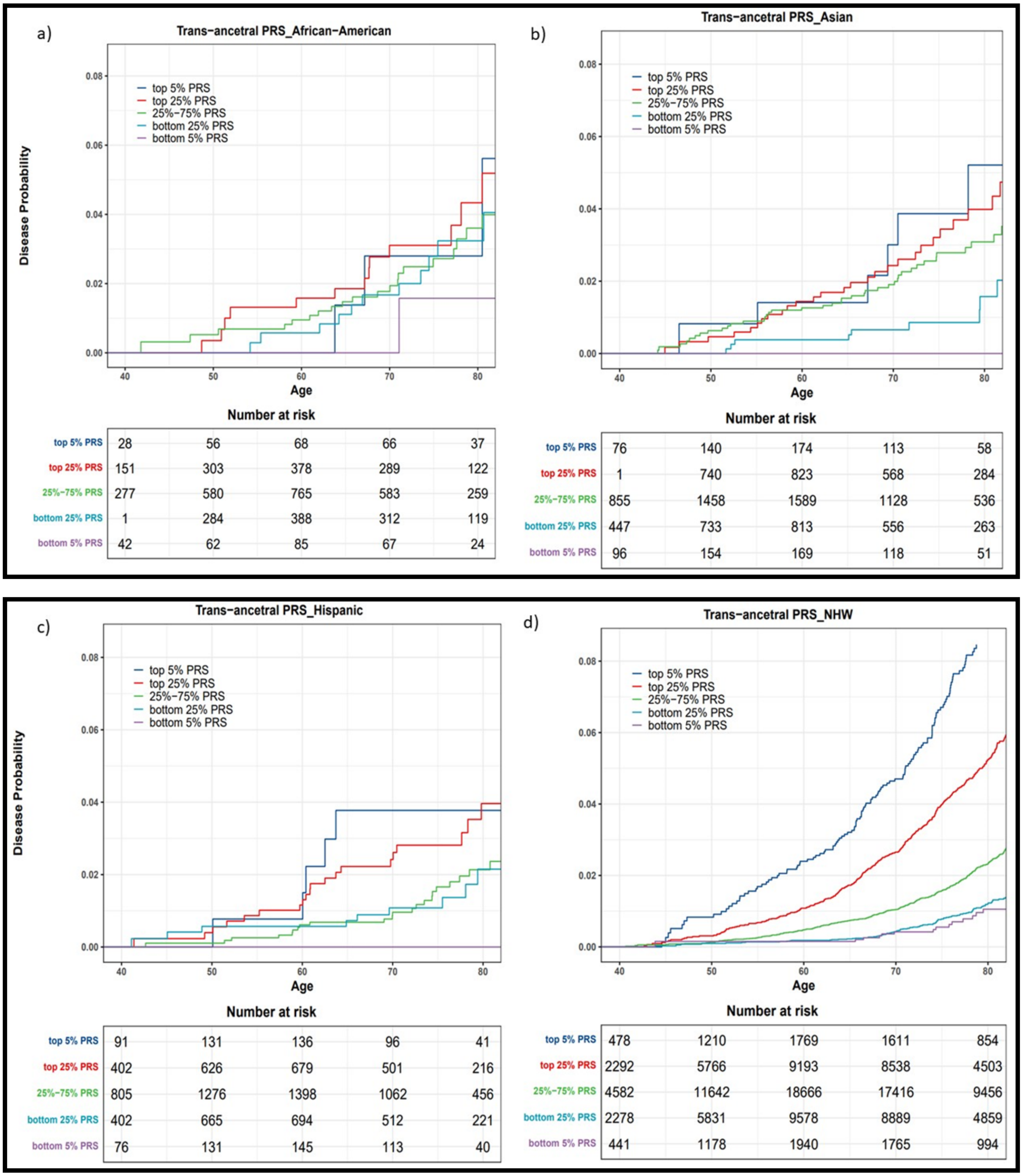

Supplementary Note 1

UK: At the Institute of Cancer Research, this work was supported by Cancer Research UK (C1298/A25514). Additional support was provided by the National Cancer Research Network. In Edinburgh, the work was supported by Programme Grant funding from Cancer Research UK (C348/A12076 to MGD, C6199/A16459 to IPMT), EU ERC Advanced Grant EVOCAN, and the infrastructure and staffing of the Edinburgh CRUK Cancer Research Centre. CFR was supported by a Marie Sklodowska-Curie Intra-European Fellowship Action (IEF-301077) for the INTERMPHEN project and received considerable help from many staff in the Department of Endoscopy at the John Radcliffe Hospital in Oxford. SEB is supported by an MRC Clinical Research Training Fellowship (MR/P001106/1). We are grateful to many colleagues within UK Clinical Genetics Departments (for CORGI) and to many collaborators who participated in the VICTOR, QUASAR2 and SCOT trials. We also thank colleagues from the UK National Cancer Research Network (for NSCCG). Support from the European Union [FP7/207–2013, grant 258236], FP7 collaborative project SYSCOL, and COST Actions EuColonGene and TransColonCan are also acknowledged [BM1206 and CA17118]. COIN translational studies were supported by Tenovus Cancer Care and Cancer Research Wales. The COIN and COIN-B trials were funded by Cancer Research UK, the Medical Research Council and an unrestricted educational grant from Merck-Serono and were conducted with the support of the National Institute of Health Research Cancer Research Network. We thank the Lothian Birth Cohorts' members, investigators, research associates, and other team members. We thank the Edinburgh Clinical Research Facility (ECRF) Genetics Core, Western General Hospital, Edinburgh, for genotyping. Lothian Birth Cohorts' data collection is supported by the Disconnected Mind project (funded by Age UK), and the Biotechnology and Biological Sciences Research Council

(BBSRC, for genotyping; BB/F019394/1) and undertaken within the University of Edinburgh Centre for Cognitive Ageing and Cognitive Epidemiology (funded by the BBSRC and Medical Research Council RC as part of the LLHW [MR/K026992/1]). ET was supported by Cancer Research UK CDF (C31250/A22804). PV-S was supported by a MRC Clinical Research Training Fellowship (MR/M004007/1). Generation Scotland received core support from the Chief Scientist Office of the Scottish Government Health Directorates [CZD/16/6] and the Scottish Funding Council [HR03006] and is currently supported by the Wellcome Trust [216767/Z/19/Z]. Genotyping of the GS: SFHS samples was carried out by the Genetics Core Laboratory at the Clinical Research Facility, University of Edinburgh and was funded by the Medical Research Council UK and the Wellcome Trust (Wellcome Trust Strategic Award "STratifying Resilience and Depression Longitudinally" (STRADL) [104036/Z/14/Z]). CH was supported by an MRC Human Genetics Unit programme grant 'Quantitative traits in health and disease' (U. MC_UU_00007/10). James East and Simon Leedham were funded by the National Institute for Health Research (NIHR) Oxford Biomedical Research Centre (BRC). The views expressed are not necessarily those of the NHS, the NIHR or the Department of Health. We acknowledge use of genotype data from the British 1958 Birth Cohort DNA collection, which was funded by the Medical Research Council Grant G0000934 and the Wellcome Trust Grant 068545/Z/02. A full list of the investigators who contributed to the generation of the data is available from http://www.wtccc.org.uk.

Finland: In Finland, this work was supported by grants from the Academy of Finland [Finnish Center of Excellence Program 2012–2017, 250345 and 2018–2025, 312041], the Jane and Aatos Erkko Foundation, the Finnish Cancer Society [also a personal grant to K.P.], the Sigrid Juselius Foundation, SYSCOL, the Nordic Information for Action eScience Center (NIASC), the Nordic

Center of Excellence financed by NordForsk [project 62721, personal grant to K.P.] and State Research Funding of Kuopio University Hospital [B1401]. We acknowledge the computational resources provided by the ELIXIR node, hosted at the CSC–IT Center for Science, Finland, and funded by the Academy of Finland [grants 271642 and 263164], the Ministry of Education and Culture, Finland. V.S. was supported by the Finnish Academy [grant number 139635] and the Finnish Foundation for Cardiovascular Research. J.-P.M. was funded by The Finnish Cancer Foundation and The Jane and Aatos Erkko Foundation. Sample collection and genotyping in the Finnish Twin Cohort has been supported by the Wellcome Trust Sanger Institute, ENGAGE—European Network for Genetic and Genomic Epidemiology, FP7-HEALTH-F4–2007; [grant agreement number 201413], the National Institute of Alcohol Abuse and Alcoholism [grants AA-12502 and AA-00145; to R.J.R. and K02AA018755 to D.M.D.] and the Academy of Finland [grants 100499, 205585, 265240 and 263278 to J.K.].

Croatia: The Croatian study was supported through the 10,001 Dalmatians Project, and institutional support of University Hospital for Tumours, Sestre Milosrdnice University Hospital Center.

BCAC: The BCAC study would not have been possible without the contributions of the following: Manjeet K. Bolla, Qin Wang, Kyriaki Michailidou and Joe Dennis. BCAC is funded by Cancer Research UK (C1287/A10118, C1287/A16563). For the BBCS study, we thank Eileen Williams, Elaine Ryder-Mills, Kara Sargus. The BBCS is funded by Cancer Research UK and Breast Cancer Now and acknowledges NHS funding to the National Institute of Health Research (NIHR) Biomedical Research Centre (BRC) and the National Cancer Research Network (NCRN). We thank the participants and the investigators of EPIC (European Prospective Investigation into Cancer and Nutrition). The coordination of EPIC is financially supported by

the European Commission (DG-SANCO) and the International Agency for Research on Cancer. The national cohorts are supported by: Ligue Contre le Cancer, Institut Gustave Roussy, Mutuelle Générale de l'Education Nationale, Institut National de la Santé et de la Recherche Médicale (INSERM) (France); German Cancer Aid, German Cancer Research Center (DKFZ), Federal Ministry of Education and Research (BMBF) (Germany); the Hellenic Health Foundation, the Stavros Niarchos Foundation (Greece); Associazione Italiana per la Ricerca sul Cancro-AIRC-Italy and National Research Council (Italy); Dutch Ministry of Public Health, Welfare and Sports (VWS), Netherlands Cancer Registry (NKR), LK Research Funds, Dutch Prevention Funds, Dutch ZON (Zorg Onderzoek Nederland), World Cancer Research Fund (WCRF), Statistics Netherlands (The Netherlands); Health Research Fund (FIS), PI13/00061 to Granada, PI13/01162 to EPIC-Murcia, Regional Governments of Andalucía, Asturias, Basque Country, Murcia and Navarra, ISCIII RETIC (RD06/0020) (Spain); Cancer Research UK (14136 to EPIC-Norfolk; C570/A16491 and C8221/A19170 to EPIC-Oxford), Medical Research Council (1000143 to EPIC-Norfolk, MR/M012190/1 to EPIC-Oxford) (United Kingdom). We thank the SEARCH and EPIC teams, which were funded by a programme grant from Cancer Research UK (C490/A10124) and supported by the UK NIHR BRC at the University of Cambridge. We thank Breast Cancer Now and the Institute of Cancer Research (ICR) for support and funding of the UKBGS, and the study participants, study staff, and the doctors, nurses and other health-care providers and health information sources who have contributed to the study. PRACTICAL: Genotyping of the PRACTICAL consortium OncoArray was funded by the US National Institutes of Health (NIH) [U19 CA 148537 for ELucidating associations Involved in Prostate cancer SuscEptibility (ELLIPSE) project and X01HG007492 to the Center for Inherited Disease Research (CIDR) under contract number HHSN268201200008I]. Additional analytic

support was provided by NIH NCI U01 CA188392 (PI: Schumacher). The PRACTICAL consortium was supported by Cancer Research UK Grants C5047/A7357, C1287/A10118, C1287/A16563, C5047/A3354, C5047/A10692, C16913/A6135, European Commission's Seventh Framework Programme grant agreement n° 223175 (HEALTH-F2–2009–223175), and The National Institute of Health (NIH) Cancer Post-Cancer GWAS initiative grant: No. 1 U19 CA 148537–01 (the GAME-ON initiative). We would also like to thank the following for funding support: The Institute of Cancer Research and The Everyman Campaign, The Prostate Cancer Research Foundation, Prostate Research Campaign UK (now Prostate Action), The Orchid Cancer Appeal, The National Cancer Research Network UK, The National Cancer Research Institute (NCRI) UK. We are grateful for support of NIHR funding to the NIHR Biomedical Research Centre at The Institute of Cancer Research and The Royal Marsden NHS Foundation Trust, the Spanish Instituto de Salud Carlos III (ISCIII) an initiative of the Spanish Ministry of Economy and Innovation (Spain), and the Xunta de Galicia (Spain). The APBC BioResource, which forms part of the PRACTICAL consortium, consists of the following members: Wayne Tilley, Gail Risbridger, Renea Taylor, Lisa Horvath, Vanessa Hayes, Lisa Butler, Trina Yeadon, Allison Eckert, Anne-Maree Haynes, Melissa Papargiris.

LD hub data: We gratefully acknowledge all the studies and databases that made GWAS summary data available through LD Hub database: ADIPOGen (Adiponectin genetics consortium), C4D (Coronary Artery Disease Genetics Consortium), CARDIoGRAM (Coronary ARtery DIsease Genome wide Replication and Meta-analysis), CKDGen (Chronic Kidney Disease Genetics consortium), dbGAP (database of Genotypes and Phenotypes), DIAGRAM (DIAbetes Genetics Replication And Meta-analysis), ENIGMA (Enhancing Neuro Imaging Genetics through Meta Analysis), EAGLE (EArly Genetics & Lifecourse Epidemiology Eczema

Consortium, excluding 23andMe), EGG (Early Growth Genetics Consortium), GABRIEL (A Multidisciplinary Study to Identify the Genetic and Environmental Causes of Asthma in the European Community), GCAN (Genetic Consortium for Anorexia Nervosa), GEFOS (GEnetic Factors for OSteoporosis Consortium), GIANT (Genetic Investigation of ANthropometric Traits), GIS (Genetics of Iron Status consortium), GLGC (Global Lipids Genetics Consortium), GPC (Genetics of Personality Consortium), GUGC (Global Urate and Gout consortium), HaemGen (haematological and platelet traits genetics consortium), HRgene (Heart Rate consortium), IIBDGC (International Inflammatory Bowel Disease Genetics Consortium), ILCCO (International Lung Cancer Consortium), IMSGC (International Multiple Sclerosis Genetic Consortium), MAGIC (Meta-Analyses of Glucose and Insulin-related traits Consortium), MESA (Multi-Ethnic Study of Atherosclerosis), PGC (Psychiatric Genomics Consortium), Project MinE consortium, ReproGen (Reproductive Genetics Consortium), SSGAC (Social Science Genetics Association Consortium) and TAG (Tobacco and Genetics Consortium), TRICL (Transdisciplinary Research in Cancer of the Lung consortium), UK Biobank. We gratefully acknowledge the contributions of Alkes Price (the systemic lupus erythematosus GWAS and primary biliary cirrhosis GWAS) and Johannes Kettunen (lipids metabolites GWAS).

The work at Vanderbilt University Medical Center was supported by U.S. NIH grants R01CA188214, R37CA070867, UM1CA182910, R01CA124558, R01CA158473, and R01CA148667, as well as Anne Potter Wilson Chair funds from the Vanderbilt University School of Medicine. Sample preparation and genotyping assays at Vanderbilt University were conducted at the Survey and Biospecimen Shared Resources and Vanderbilt Microarray Shared Resource, supported in part by the Vanderbilt-Ingram Cancer Center (P30CA068485). Statistical

analyses were performed on servers maintained by the Advanced Computing Center for Research and Education (ACCRE) at Vanderbilt University (Nashville, TN). Studies (listed with grant support) participating in the Asia Colorectal Cancer Consortium include the Shanghai Women's Health Study (US NIH, R37CA070867, UM1CA182910), the Shanghai Men's Health Study (US NIH, R01CA082729, UM1CA173640), the Shanghai Breast and Endometrial Cancer Studies (US NIH, R01CA064277 and R01CA092585; contributing only controls), the Shanghai Colorectal Cancer Study 3 (US NIH, R37CA070867, R01CA188214 and Anne Potter Wilson Chair funds), the Guangzhou Colorectal Cancer Study (National Key Scientific and Technological Project, 2011ZX09307-001-04; the National Basic Research Program, 2011CB504303, contributing only controls, the Natural Science Foundation of China, 81072383, contributing only controls), the Hwasun Cancer Epidemiology Study–Colon and Rectum Cancer (HCES-CRC; grants from Chonnam National University Hwasun Hospital Biomedical Research Institute, HCRI18007), the Japan BioBank Colorectal Cancer Study (grant from the Ministry of Education, Culture, Sports, Science and Technology of the Japanese government), the Aichi Colorectal Cancer Study (Grant-in-Aid for Cancer Research, grant for the Third Term Comprehensive Control Research for Cancer and Grants-in-Aid for Scientific Research from the Japanese Ministry of Education, Culture, Sports, Science and Technology, 17015018 and 221S0001), the Korea-NCC (National Cancer Center) Colorectal Cancer Study (Basic Science Research Program through the National Research Foundation of Korea, 2010-0010276 and 2013R1A1A2A10008260; National Cancer Center Korea, 0910220), and the KCPS-II Colorectal Cancer Study (National R&D Program for Cancer Control, 1631020; Seoul R&D Program, 10526).

GECCO: Genetics and Epidemiology of Colorectal Cancer Consortium: National Cancer Institute, National Institutes of Health, U.S. Department of Health and Human Services (R01 CA244588, U01 CA164930, U01 CA137088, R01 CA059045, R01CA201407, R01CA206279, U01CA261339, R01 CA185094). Genotyping services were provided by the Center for Inherited Disease Research (CIDR) contract number HHSN268201200008I. This research was funded in part through the NIH/NCI Cancer Center Support Grant P30 CA015704. Scientific Computing Infrastructure at Fred Hutch funded by ORIP grant S10OD028685.

ASTERISK: A Hospital Clinical Research Program (PHRC-BRD09/C) from the University Hospital Center of Nantes (CHU de Nantes) and supported by the Regional Council of Pays de la Loire, the Groupement des Entreprises Françaises dans la Lutte contre le Cancer (GEFLUC), the Association Anne de Bretagne Génétique and the Ligue Régionale Contre le Cancer (LRCC). We are very grateful to Dr. Bruno Buecher without whom this project would not have existed. We also thank all those who agreed to participate in this study, including the patients and the healthy control persons, as well as all the physicians, technicians and students.

ATBC: The Alpha-Tocopherol, Beta-Carotene Cancer Prevention Study is supported by the Intramural Research Program of the U.S. National Cancer Institute, National Institutes of Health, Department of Health and Human Services.

Black Women's Health Study (BWHS); funding from the National Cancer Institute (R01CA058420, R01CA098663, and U01CA164974).

CLUE II funding was from the National Cancer Institute (U01 CA86308, Early Detection Research Network; P30 CA006973), National Institute on Aging (U01 AG18033), and the American Institute for Cancer Research. The content of this publication does not necessarily

reflect the views or policies of the Department of Health and Human Services, nor does mention of trade names, commercial products, or organizations imply endorsement by the US government. Maryland Cancer Registry (MCR) Cancer data was provided by the Maryland Cancer Registry, Center for Cancer Prevention and Control, Maryland Department of Health, with funding from the State of Maryland and the Maryland Cigarette Restitution Fund. The collection and availability of cancer registry data is also supported by the Cooperative Agreement NU58DP006333, funded by the Centers for Disease Control and Prevention. Its contents are solely the responsibility of the authors and do not necessarily represent the official views of the Centers for Disease Control and Prevention or the Department of Health and Human Services.

ColoCare: This work was supported by the National Institutes of Health (grant numbers R01 CA189184 (Li/Ulrich), U01 CA206110 (Ulrich/Li/Siegel/Figueireido/Colditz), 2P30CA015704-40 (Gilliland), R01 CA207371 (Ulrich/Li), P30-CA076292 (Cleveland)), the Matthias Lackas-Foundation, the German Consortium for Translational Cancer Research, and the EU TRANSCAN initiative.

The Colon Cancer Family Registry (CCFR, www.coloncfr.org) is supported in part by funding from the National Cancer Institute (NCI), National Institutes of Health (NIH) (award U01 CA167551). Support for case ascertainment was provided in part from the Surveillance, Epidemiology, and End Results (SEER) Program and the following U.S. state cancer registries: AZ, CO, MN, NC, NH; and by the Victoria Cancer Registry (Australia) and Ontario Cancer Registry (Canada). The CCFR Set-1 (Illumina 1M/1M-Duo) and Set-2 (Illumina Omni1-Quad) scans were supported by NIH awards U01 CA122839 and R01 CA143237 (to GC). The CCFR Set-3 (Affymetrix Axiom CORECT Set array) was supported by NIH award U19 CA148107 and

R01 CA81488 (to SBG). The CCFR Set-4 (Illumina OncoArray 600K SNP array) was supported by NIH award U19 CA148107 (to SBG) and by the Center for Inherited Disease Research (CIDR), which is funded by the NIH to the Johns Hopkins University, contract number HHSN268201200008I. Additional funding for the OFCCR/ARCTIC was through award GL201-043 from the Ontario Research Fund (to BWZ), award 112746 from the Canadian Institutes of Health Research (to TJH), through a Cancer Risk Evaluation (CaRE) Program grant from the Canadian Cancer Society (to SG), and through generous support from the Ontario Ministry of Research and Innovation. The SFCCR Illumina HumanCytoSNP array was supported in part through NCI/NIH awards U01 CA074794 (to JDP) and /U24 CA074794 and R01 CA076366 (to PAN). The content of this manuscript does not necessarily reflect the views or policies of the NCI, NIH or any of the collaborating centers in the Colon Cancer Family Registry (CCFR), nor does mention of trade names, commercial products, or organizations imply endorsement by the US Government, any cancer registry, or the CCFR.

COLON: The COLON study is sponsored by Wereld Kanker Onderzoek Fonds, including funds from grant 2014/1179 as part of the World Cancer Research Fund International Regular Grant Programme, by Alpe d'Huzes and the Dutch Cancer Society (UM 2012–5653, UW 2013-5927, UW2015-7946), and ERA-NET on Translational Cancer Research (TRANSCAN: Dutch Cancer Society (UW2013-6397, UW2014-6877) and the Netherlands Organization for Health Research and Development (ZonMW), the Netherlands) and the Regio Deal Foodvalley (162135). The NQplus study is sponsored by a ZonMW investment grant (98-10030); by PREVIEW, the project PREVention of diabetes through lifestyle intervention and population studies in Europe and around the World (PREVIEW) project which received funding from the European Union Seventh Framework Programme (FP7/2007–2013) under grant no. 312057; by funds from TI

Food and Nutrition (cardiovascular health theme), a public–private partnership on pre-competitive research in food and nutrition; and by FOODBALL, the Food Biomarker Alliance, a project from JPI Healthy Diet for a Healthy Life.

Colorectal Cancer Transdisciplinary (CORECT) Study: The CORECT Study was supported by the National Cancer Institute, National Institutes of Health (NCI/NIH), U.S. Department of Health and Human Services (grant numbers U19 CA148107, R01 CA81488, P30 CA014089, R01 CA197350; P01 CA196569; R01 CA201407) and National Institutes of Environmental Health Sciences, National Institutes of Health (grant number T32 ES013678).

CORSA: The CORSA study was funded by Austrian Research Funding Agency (FFG) BRIDGE (grant 829675, to A. G.), the "Herzfeldersche Familienstiftung" (grant to A.G.) and was supported by COST Action BM1206 and CA17118.

CPS-II: The American Cancer Society funds the creation, maintenance, and updating of the Cancer Prevention Study-II (CPS-II) cohort. The study protocol was approved by the institutional review boards of Emory University, and those of participating registries as required. The authors assume full responsibility for all analyses and interpretation of results. The views expressed here are those of the authors and do not necessarily represent the American Cancer Society or the American Cancer Society – Cancer Action Network.

CRCGEN: Colorectal Cancer Genetics & Genomics, Spanish study was supported by Instituto de Salud Carlos III, co-funded by FEDER funds –a way to build Europe– (grants PI14-613 and PI09-1286), Agency for Management of University and Research Grants (AGAUR) of the Catalan Government (grant 2017SGR723), Junta de Castilla y León (grant LE22A10-2), the Spanish Association Against Cancer (AECC) Scientific Foundation grant GCTRA18022MORE and the Consortium for Biomedical Research in Epidemiology and Public Health (CIBERESP),

action Genrisk. Sample collection of this work was supported by the Xarxa de Bancs de Tumors de Catalunya sponsored by Pla Director d'Oncología de Catalunya (XBTC), Plataforma Biobancos PT13/0010/0013 and ICOBIOBANC, sponsored by the Catalan Institute of Oncology. We thank CERCA Programme, Generalitat de Catalunya for institutional support.
Czech Republic CCS: This work was supported by the Czech Science Foundation (21-27902S and 22-05942S) and by the Grant Agency of the Ministry of Health of the Czech Republic (grants NU21-03-00506).
DACHS: This work was supported by the German Research Council (BR 1704/6-1, BR 1704/6-3, BR 1704/6-4, CH 117/1-1, HO 5117/2-1, HE 5998/2-1, KL 2354/3-1, RO 2270/8-1 and BR 1704/17-1), the Interdisciplinary Research Program of the National Center for Tumor Diseases (NCT), Germany, and the German Federal Ministry of Education and Research (01KH0404, 01ER0814, 01ER0815, 01ER1505A and 01ER1505B).
DALS: National Institutes of Health (R01 CA48998 to M. L. Slattery).
EDRN: This work is funded and supported by the NCI, EDRN Grant (U01 CA 84968-06).
eMERGE Network (Phase III): This phase of the eMERGE Network was initiated and funded by the NHGRI through the following grants: U01HG008657 (University of Washington Medical Center); U01HG008685 (Brigham and Women's Hospital); U01HG008672 (Vanderbilt University Medical Center); U01HG008666 (Cincinnati Children's Hospital Medical Center); U01HG006379 (Mayo Clinic); U01HG008679 (Geisinger Clinic); U01HG008680 (Columbia University Health Sciences); U01HG008684 (Children's Hospital of Philadelphia); U01HG008673 (Northwestern University); U01HG008701 (Vanderbilt University Medical Center serving as the Coordinating Center); U01HG008676 (Partners Healthcare/Broad Institute); U01HG008664 (Baylor College of Medicine); and U54MD007593 (Meharry Medical

College). The eMERGE imputed merged Phase I and Phase II dataset: U01HG004438 (CIDR) and U01HG004424 (the Broad Institute) serving as Genotyping Centers.
EPIC: The coordination of EPIC is financially supported by the European Commission (DGSANCO) and the International Agency for Research on Cancer. The national cohorts are supported by Danish Cancer Society (Denmark); Ligue Contre le Cancer, Institut Gustave Roussy, Mutuelle Générale de l'Education Nationale, Institut National de la Santé et de la Recherche Médicale (INSERM) (France); German Cancer Aid, German Cancer Research Center (DKFZ), Federal Ministry of Education and Research (BMBF), Deutsche Krebshilfe, Deutsches Krebsforschungszentrum and Federal Ministry of Education and Research (Germany); the Hellenic Health Foundation (Greece); Associazione Italiana per la Ricerca sul Cancro-AIRCItaly and National Research Council (Italy); Dutch Ministry of Public Health, Welfare and Sports (VWS), Netherlands Cancer Registry (NKR), LK Research Funds, Dutch Prevention Funds, Dutch ZON (Zorg Onderzoek Nederland), World Cancer Research Fund (WCRF), Statistics Netherlands (The Netherlands); ERC-2009-AdG 232997 and Nordforsk, Nordic Centre of Excellence programme on Food, Nutrition and Health (Norway); Health Research Fund (FIS), PI13/00061 to Granada, PI13/01162 to EPIC-Murcia, Regional Governments of Andalucía, Asturias, Basque Country, Murcia and Navarra, ISCIII RETIC (RD06/0020) (Spain); Swedish Cancer Society, Swedish Research Council, Region Skåne and Region Västerbotten (Sweden); Cancer Research UK (14136 to EPIC-Norfolk; C570/A16491 and C8221/A19170 to EPIC-Oxford), Medical Research Council (1000143 to EPIC-Norfolk, MR/M012190/1 to EPICOxford) (United Kingdom).
The EPIC-Norfolk study (https://doi.org/10.22025/2019.10.105.00004) has received funding from the Medical Research Council (MR/N003284/1 and MC-UU_12015/1) and Cancer

Research UK (C864/A14136). The genetics work in the EPIC-Norfolk study was funded by the Medical Research Council (MC_PC_13048). Metabolite measurements in the EPIC-Norfolk study were supported by the MRC Cambridge Initiative in Metabolic Science (MR/L00002/1) and the Innovative Medicines Initiative Joint Undertaking under EMIF grant agreement no. 115372.

EPICOLON: This work was supported by grants from Fondo de Investigación Sanitaria/FEDER (PI08/0024, PI08/1276, PS09/02368, PI11/00219, PI11/00681, PI14/00173, PI14/00230, PI17/00509, 17/00878, PI20/00113, PI20/00226, Acción Transversal de Cáncer), Xunta de Galicia (PGIDIT07PXIB9101209PR), Ministerio de Economia y Competitividad (SAF07-64873, SAF 2010-19273, SAF2014-54453R), Fundación Científica de la Asociación Española contra el Cáncer (GCB13131592CAST, PRYGN211085CAST), Beca Grupo de Trabajo "Oncología" AEG (Asociación Española de Gastroenterología), Fundación Privada Olga Torres, FP7 CHIBCHA Consortium, Agència de Gestió d'Ajuts Universitaris i de Recerca (AGAUR, Generalitat de Catalunya, 2014SGR135, 2014SGR255, 2017SGR21, 2017SGR653), Catalan Tumour Bank Network (Pla Director d'Oncologia, Generalitat de Catalunya), PERIS (SLT002/16/00398, Generalitat de Catalunya), Marató TV3 (202008-10), CERCA Programme (Generalitat de Catalunya) and COST Actions BM1206 and CA17118. CIBERehd is funded by the Instituto de Salud Carlos III.

ESTHER/VERDI. This work was supported by grants from the Baden-Württemberg Ministry of Science, Research and Arts and the German Cancer Aid.

GERA: Data used in this study were generated by the Kaiser Permanente Research Program on Genes, Environment, and Health (RPGEH), including the Genetic Epidemiology Research on Adult Health and Aging (GERA) data. The RPGEH has been funded by the National Institutes of

Health [RC2 AG036607], the Robert Wood Johnson Foundation, the Wayne and Gladys Valley Foundation, The Ellison Medical Foundation, and the Kaiser Permanente Community Benefit Program. This study has also been supported in part by a grant from the National Cancer Institute [R01CA206279 and UM1CA222035].Harvard cohorts (HPFS, NHS, PHS): HPFS is supported by the National Institutes of Health (P01 CA055075, UM1 CA167552, U01 CA167552, R01 CA137178, R01 CA151993, and R35 CA197735), NHS by the National Institutes of Health (R01 CA137178, P01 CA087969, UM1 CA186107, R01 CA151993, and R35 CA197735) and PHS by the National Institutes of Health (R01 CA042182).

Hawaii Adenoma Study: NCI grants R01 CA72520.

HCES-CRC: the Hwasun Cancer Epidemiology Study–Colon and Rectum Cancer (HCES-CRC; grants from Chonnam National University Hwasun Hospital, HCRI15011-1).

IARC: disclaimer: where authors are identified as personnel of the INternational Agency for Research on Cancer/World Health Organization, the authors alone are responsible for the views expressed in this article and they do not necessarily represent the decisions, policy or views of the International Agency for Research on Cancer/World Health Organization.

JPHC: The Japan Public Health Center-based Prospective study was supported by the National Cancer Center Research and Development Fund (23-A- 31[toku], 26-A-2, 29-A-4, 2020-J-4) (since 2011) and a Grant-in-Aid for Cancer Research from the Ministry of Health, Labour and Welfare of Japan (from 1989 to 2010)

Kentucky: This work was supported by the following grant support: Clinical Investigator Award from Damon Runyon Cancer Research Foundation (CI-8); NCI R01CA136726.

LCCS: The Leeds Colorectal Cancer Study was funded by the Food Standards Agency and Cancer Research UK Programme Award (C588/A19167).

Melbourne Collaborative Cohort Study (MCCS) cohort recruitment was funded by VicHealth and Cancer Council Victoria. The MCCS was further augmented by Australian National Health and Medical Research Council grants 209057, 396414 and 1074383 and by infrastructure provided by Cancer Council Victoria. Cases and their vital status were ascertained through the Victorian Cancer Registry and the Australian Institute of Health and Welfare, including the National Death Index and the Australian Cancer Database.

Multiethnic Cohort (MEC) Study: National Institutes of Health (R37 CA54281, P01 CA033619, R01 CA063464 and U01 CA164973).

MECC: This work was supported by the National Institutes of Health, U.S. Department of Health and Human Services (R01 CA81488 to SBG and GR).

MSKCC: The work at Sloan Kettering in New York was supported by the Robert and Kate Niehaus Center for Inherited Cancer Genomics and the Romeo Milio Foundation. Moffitt: This work was supported by funding from the National Institutes of Health (grant numbers R01 CA189184, P30 CA076292), Florida Department of Health Bankhead-Coley Grant 09BN-13, and the University of South Florida Oehler Foundation. Moffitt contributions were supported in part by the Total Cancer Care Initiative, Collaborative Data Services Core, and Tissue Core at the H. Lee Moffitt Cancer Center & Research Institute, a National Cancer Institute-designated Comprehensive Cancer Center (grant number P30 CA076292).

NCCCS I & II: We acknowledge funding support for this project from the National Institutes of Health, R01 CA66635 and P30 DK034987.

NFCCR: This work was supported by an Interdisciplinary Health Research Team award from the Canadian Institutes of Health Research (CRT 43821); the National Institutes of Health, U.S. Department of Health and Human Services (U01 CA74783); and National Cancer Institute of

Canada grants (18223 and 18226). The authors wish to acknowledge the contribution of Alexandre Belisle and the genotyping team of the McGill University and Génome Québec Innovation Centre, Montréal, Canada, for genotyping the Sequenom panel in the NFCCR samples. Funding was provided to Michael O. Woods by the Canadian Cancer Society Research Institute.

NSHDS: Swedish Research Council (VR 2017-01737) in part through support to Biobank Sweden (VR 2017-00650); Swedish Cancer Society (CAN 2017/581); Cutting-Edge Research Grant and other grants from Region Västerbotten (VLL-841671, VLL-833291); Knut and Alice Wallenberg Foundation; Lion's Cancer Research Foundation at Umeå University; the Cancer Research Foundation in Northern Sweden; and the Faculty of Medicine, Umeå University, Umeå, Sweden.

OSUMC: OCCPI funding was provided by Pelotonia and HNPCC funding was provided by the NCI (CA16058 and CA67941).

PLCO: Intramural Research Program of the Division of Cancer Epidemiology and Genetics and supported by contracts from the Division of Cancer Prevention, National Cancer Institute, NIH, DHHS. Funding was provided by National Institutes of Health (NIH), Genes, Environment and Health Initiative (GEI) Z01 CP 010200, NIH U01 HG004446, and NIH GEI U01 HG 004438.

SEARCH: The University of Cambridge has received salary support in respect of PDPP from the NHS in the East of England through the Clinical Academic Reserve; Cancer Research UK (C490/A16561); the UK National Institute for Health Research Biomedical Research Centre at the University of Cambridge.

SELECT: Research reported in this publication was supported in part by the National Cancer Institute of the National Institutes of Health under Award Numbers U10 CA37429 (CD Blanke),

and UM1 CA182883 (CM Tangen/IM Thompson). The content is solely the responsibility of the authors and does not necessarily represent the official views of the National Institutes of Health.

SMS and REACH: This work was supported by the National Cancer Institute (grant P01 CA074184 to J.D.P. and P.A.N., grants R01 CA097325, R03 CA153323, and K05 CA152715 to P.A.N., and the National Center for Advancing Translational Sciences at the National Institutes of Health (grant KL2 TR000421 to A.N.B.-H.)

The Swedish Low-risk Colorectal Cancer Study: The study was supported by grants from the Swedish research council; K2015-55X-22674-01-4, K2008-55X-20157-03-3, K2006-72X-20157-01-2 and the Stockholm County Council (ALF project).

Swedish Mammography Cohort and Cohort of Swedish Men: This work is supported by the Swedish Research Council /Infrastructure grant, the Swedish Cancer Foundation, and the Karolinska Institute´s Distinguished Professor Award to Alicja Wolk.

UK Biobank: This research has been conducted using the UK Biobank Resource under Application Number 8614

VITAL: National Institutes of Health (K05 CA154337).

WHI: The WHI program is funded by the National Heart, Lung, and Blood Institute, National Institutes of Health, U.S. Department of Health and Human Services through contracts HHSN268201100046C, HHSN268201100001C, HHSN268201100002C, HHSN268201100003C, HHSN268201100004C, and HHSN271201100004C.

The Asia Colorectal Cancer Consortium is primarily funded by R01 CA188214 from US National Institutes of Health. Studies (listed with grant support) participating in the Asia Colorectal Cancer Consortium include the Shanghai Women's Health Study (US NIH, R37CA070867, UM1CA182910), the Shanghai Men's Health Study (US NIH, R01CA082729,

UM1CA173640), the Shanghai Breast and Endometrial Cancer Studies (US NIH, R01CA064277 and R01CA092585; contributing only controls), the Shanghai Colorectal Cancer Study 3 (US NIH, R37CA070867,R01CA188214 and Anne Potter Wilson Chair funds), the Guangzhou Colorectal Cancer Study (National Key Scientific and Technological Project, 2011ZX09307-001-04; the National Basic Research Program, 2011CB504303, contributing only controls, the Natural Science Foundation of China, 81072383, contributing only controls), the Hwasun Cancer Epidemiology Study–Colon and Rectum Cancer (HCES-CRC; grants from Chonnam National University Hwasun Hospital Biomedical Research Institute,HCRI18007), the Japan BioBank Colorectal Cancer Study (grant from the Ministry of Education, Culture, Sports, Science and Technology of the Japanese government), the Aichi Colorectal Cancer Study (Grant-in-Aid for Cancer Research, grant for the Third Term Comprehensive Control Research for Cancer and Grants-in-Aid for Scientific Research from the Japanese Ministry of Education, Culture, Sports, Science and Technology, 17015018 and 221S0001), the Korea-NCC (National Cancer Center) Colorectal Cancer Study (Basic Science Research Program through the National Research Foundation of Korea, 2010-0010276 and 2013R1A1A2A10008260; National Cancer Center Korea, 0910220 and 1710882), the KCPS-II Colorectal Cancer Study (National R&D Program for Cancer Control, 1631020; Seoul R&D Program, 10526).

CCFR: The Colon CFR graciously thanks the generous contributions of their study participants, dedication of study staff, and the financial support from the U.S. National Cancer Institute, without which this important registry would not exist. The authors would like to thank the study participants and staff of the Seattle Colon Cancer Family Registry and the Hormones and Colon Cancer study (CORE Studies).

CLUE II: We thank the participants of Clue II and appreciate the continued efforts of the staff at the Johns Hopkins George W. Comstock Center for Public Health Research and Prevention in the conduct of the Clue II Cohort Study.
COLON and NQplus: the authors would like to thank all participants, the involved co-workers in the participating hospitals, and the COLON and NQplus investigators at Wageningen University & Research.
CORSA: We kindly thank all individuals who agreed to participate in the CORSA study. Furthermore, we thank all cooperating physicians and students and the Biobank Graz of the Medical University of Graz.
CPS-II: The authors thank the CPS-II participants, and to each member of the study and biospecimen management group. The authors would like to acknowledge the contribution to this study from central cancer registries supported through the Centers for Disease Control and Prevention National Program of Cancer Registries, and cancer registries supported by the National Cancer Institute Surveillance Epidemiology and End Results program.
Czech Republic CCS: We are thankful to all clinicians in major hospitals in the Czech Republic, without whom the study would not be practicable. We are also sincerely grateful to all patients participating in this study.
DACHS: We thank all participants and cooperating clinicians, and Ute Handte-Daub, Utz Benscheid, Muhabbet Celik and Ursula Eilber for excellent technical assistance.
EDRN: We acknowledge all the following contributors to the development of the resource: University of Pittsburgh School of Medicine, Department of Gastroenterology, Hepatology and Nutrition: Lynda Dzubinski; University of Pittsburgh School of Medicine, Department of

Pathology: Michelle Bisceglia; and University of Pittsburgh School of Medicine, Department of Biomedical Informatics.
EPIC: Where authors are identified as personnel of the International Agency for Research on Cancer/World Health Organization, the authors alone are responsible for the views expressed in this article and they do not necessarily represent the decisions, policy or views of the International Agency for Research on Cancer/World Health Organization.
The EPIC-Norfolk study: we are grateful to all the participants who have been part of the project and to the many members of the study teams at the University of Cambridge who have enabled this research.
EPICOLON: We are sincerely grateful to all patients participating in this study who were recruited as part of the EPICOLON project. We acknowledge the Spanish National DNA Bank, Biobank of Hospital Clínic–IDIBAPS and Biobanco Vasco for the availability of the samples. The work was carried out (in part) at the Esther Koplowitz Centre, Barcelona.
Harvard cohorts (HPFS, NHS, PHS): The study protocol was approved by the institutional review boards of the Brigham and Women's Hospital and Harvard T.H. Chan School of Public Health, and those of participating registries as required. We would like to thank the participants and staff of the HPFS, NHS and PHS for their valuable contributions as well as the following state cancer registries for their help: AL, AZ, AR, CA, CO, CT, DE, FL, GA, ID, IL, IN, IA, KY, LA, ME, MD, MA, MI, NE, NH, NJ, NY, NC, ND, OH, OK, OR, PA, RI, SC, TN, TX, VA, WA, WY. The authors assume full responsibility for analyses and interpretation of these data.
Interval: A complete list of the investigators and contributors to the INTERVAL trial is provided in reference (32). The academic coordinating centre would like to thank blood donor centre staff and blood donors for participating in the INTERVAL trial.

Kentucky: We would like to acknowledge the staff at the Kentucky Cancer Registry.

LCCS: We acknowledge the contributions of co-investigators: David Forman, Roland Wolf, Jennifer Barrett, Robin Waxman, Gillian Smith and Emma Northwood in conducting this study as well as all the interviewers.

NCCCS I & II: We would like to thank the study participants, and the NC Colorectal Cancer Study staff.

NSHDS investigators thank the Biobank Research Unit at Umeå University, the Västerbotten Intervention Programme, the Northern Sweden MONICA study and Region Västerbotten for providing data and samples and acknowledge the contribution from Biobank Sweden, supported by the Swedish Research Council (VR 2017-00650).

PLCO: The authors thank the PLCO Cancer Screening Trial screening center investigators and the staff from Information Management Services Inc and Westat Inc. Most importantly, we thank the study participants for their contributions that made this study possible.

SEARCH: We thank the SEARCH team.

SELECT: We thank the research and clinical staff at the sites that participated on SELECT study, without whom the trial would not have been successful. We are also grateful to the 35,533 dedicated men who participated in SELECT.

UK Biobank: This research has been conducted using the UK Biobank Resource under Application Number 8614 and 7441. We would like to thank the participants and researchers UK Biobank for their participation and acquisition of data.

WHI: The authors thank the WHI investigators and staff for their dedication, and the study participants for making the program possible. A full listing of WHI investigators can be found at:

http://www.whi.org/researchers/Documents%20%20Write%20a%20Paper/WHI%20Investigator

%20Short%20List.pdf

Supplementary References

1. Wang, H. *et al.* Trans-ethnic genome-wide association study of colorectal cancer identifies a new susceptibility locus in VTI1A. *Nat. Commun.* **5**, 4613 (2014).

2. Schmit, S. L. *et al.* Genome-wide association study of colorectal cancer in Hispanics. *Carcinogenesis* **37**, 547–556 (2016).

3. Calle, E. E. *et al.* The American Cancer Society Cancer Prevention Study II Nutrition Cohort: rationale, study design, and baseline characteristics. *Cancer* **94**, 2490–2501 (2002).

4. Hoffmann, T. J. *et al.* Next generation genome-wide association tool: design and coverage of a high-throughput European-optimized SNP array. *Genomics* **98**, 79–89 (2011).

5. Kvale, M. N. *et al.* Genotyping informatics and quality control for 100,000 subjects in the genetic epidemiology research on adult health and aging (GERA) cohort. *Genetics* **200**, 1051–1060 (2015).

6. Chisholm, R. L. At the Interface between Medical Informatics and Personalized Medicine: The eMERGE Network Experience. *Healthc. Inform. Res.* **19**, 67–68 (2013).

7. Crawford, D. C. *et al.* eMERGEing progress in genomics-the first seven years. *Front. Genet.* **5**, 184 (2014).

8. Gottesman, O. *et al.* The Electronic Medical Records and Genomics (eMERGE) Network: past, present, and future. *Genet. Med.* **15**, 761–771 (2013).

9. Stanaway, I. B. *et al.* The eMERGE genotype set of 83,717 subjects imputed to ~40 million variants genome wide and association with the herpes zoster medical record phenotype. *Genet. Epidemiol.* **43**, 63–81 (2019).

10. Ruan, Y. *et al.* Improving polygenic prediction in ancestrally diverse populations. *Nat. Genet.* **54**, 573–580 (2022).

11. Vilhjálmsson, B. J. *et al.* Modeling linkage disequilibrium increases accuracy of polygenic risk scores. *Am. J. Hum. Genet.* **97**, 576–592 (2015).

12. Privé, F., Arbel, J. & Vilhjálmsson, B. J. LDpred2: better, faster, stronger. *Bioinformatics* **36**, 5424–5431 (2020).

13. Collister, J. A., Liu, X. & Clifton, L. Calculating polygenic risk scores (PRS) in UK biobank: A practical guide for epidemiologists. *Front. Genet.* **13**, 818574 (2022).

14. Chang, C. C. *et al.* Second-generation PLINK: rising to the challenge of larger and richer datasets. *Gigascience* **4**, 7 (2015).
