# Supplementary material for: Combining Asian and European genome-wide association studies of colorectal cancer improves risk prediction across racial and ethnic populations
Source: Nat Commun. 2023 Oct 2;14:6147. doi: 10.1038/s41467-023-41819-0 (PMC10545678; doi:10.1038/s41467-023-41819-0)
Supplement: Supplementary file 3 — Description of Additional Supplementary Files [file 41467_2023_41819_MOESM3_ESM.pdf]

Description of Additional Supplementary Files

Supplementary Data 1: Description of all studies included in the GWAS analysis -- European and Asian Summary Statistics

Supplementary Data 2: SNP associations for newly and previously identified colorectal cancer risk loci
